# Distribution of active ingredients of a commercial aflatoxin biocontrol product in naturally occurring fungal communities across Kenya

**DOI:** 10.1111/1751-7915.13708

**Published:** 2020-12-18

**Authors:** Md‐Sajedul Islam, Kenneth A. Callicott, Charity Mutegi, Ranajit Bandyopadhyay, Peter J. Cotty

**Affiliations:** ^1^ School of Plant Sciences USDA‐ARS The University of Arizona Tucson AZ 85721 USA; ^2^ International Institute of Tropical Agriculture Nairobi Kenya; ^3^ College of Food Science and Engineering Ocean University of China Qingdao Shandong 266003 China

## Abstract

Human populations in Kenya are repeatedly exposed to dangerous aflatoxin levels through consumption of contaminated crops. Biocontrol with atoxigenic *Aspergillus flavus* is an effective method for preventing aflatoxin in crops. Although four atoxigenic *A. flavus* isolates (C6E, E63I, R7H and R7K) recovered from maize produced in Kenya are registered as active ingredients for a biocontrol product (Aflasafe KE01) directed at preventing contamination, natural distributions of these four genotypes prior to initiation of commercial use have not been reported. Distributions of the active ingredients of KE01 based on haplotypes at 17 SSR loci are reported. Incidences of the active ingredients and closely related haplotypes were determined in soil collected from 629 maize fields in consecutive long and short rains seasons of 2012. The four KE01 haplotypes were among the top ten most frequent. Haplotype H‐1467 of active ingredient R7K was the most frequent and widespread haplotype in both seasons and was detected in the most soils (3.8%). The four KE01 haplotypes each belonged to large clonal groups containing 27–46 unique haplotypes distributed across multiple areas and in 21% of soils. Each of the KE01 haplotypes belonged to a distinct vegetative compatibility group (VCG), and all *A. flavus* with haplotypes matching a KE01 active ingredient belonged to the same VCG as the matching active ingredient as did all *A. flavus* haplotypes differing at only one SSR locus. Persistence of the KE01 active ingredients in Kenyan agroecosystems is demonstrated by detection of identical SSR haplotypes six years after initial isolation. The data provide baselines for assessing long‐term influences of biocontrol applications in highly vulnerable production areas of Kenya.

## Graphical abstract

Distributions of the active ingredients of the aflatoxin biocontrol product Aflasafe KE01 across the most vulnerable portions of Kenya were determined with 17 SSR loci. The four active ingredient haplotypes were all among the top 10 most frequent and each belonged to both a distinct vegetative compatibility group and a distinct clonal lineage containing greater than 25 haplotypes. The data provide baselines for assessing long‐term influences of biocontrol applications in highly vulnerable production areas of Kenya.

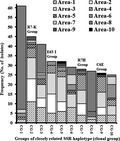

## Introduction

Several species in *Aspergillus* section *Flavi* produce aflatoxins (highly toxic, carcinogenic fungal metabolites) during infection of host crops. Aflatoxin contaminates 25% of world food crops, and 4.5 billion people in developing countries, especially in Africa and Asia, are chronically exposed to aflatoxins through consumption of staple crops (Williams *et al*., 2004). Maize is the primary staple in Kenya, a major ‘hotspot’ for aflatoxin (Mutegi *et al*., 2018) with exposure frequently documented as aflatoxin B1‐lysine adduct in serum of children (Gong *et al*., 2012) and aflatoxin M_1_ in mother’s milk (Mutegi *et al*., 2018). Indeed, Kenya repeatedly experienced epidemics of lethal aflatoxicosis since 1981 (Liu and Wu, 2010; WHO, 2011) and nearly 317 cases of acute aflatoxicosis with over 125 deaths occurred in 2004 in the Coastal and Eastern provinces of Kenya (Azziz‐Baumgartner *et al*., 2005). In a 2010, human serum aflatoxin levels in Kenyans were among the highest ever recorded (Obura, 2013) and a cross‐sectional serosurvey documented a widespread urgent need to implement interventions to minimize aflatoxin exposure (Yard *et al*., 2013).

The Centers for Disease Control and Prevention (CDC) and the Kenya Ministry of Public Health and Sanitation focused on efforts to reduce aflatoxin exposure (Mutegi *et al*., 2018). Biocontrol of aflatoxin‐producing fungi is a promising method for reducing crop contamination (Probst *et al*., 2011). Atoxigenic strains of *A. flavus* are used to alter natural fungal communities with non‐aflatoxin‐producing (atoxigenic) *A. flavus* so that aflatoxin producers are less common and the average aflatoxin‐producing potentials of fungal communities are reduced (Cotty *et al*., 2008; Bandyopadhyay *et al*., 2016). This change in the fungal community results in greatly reduced aflatoxin concentrations in crops. Atoxigenic strain‐based biocontrol has been successfully used in the United States for over two decades in a variety of crops, such as cotton (Cotty, 1994a, 1994b,1994a, 1994b; Cotty *et al*., 2007), groundnut (Dorner *et al*., 1998), pistachio (Doster *et al*., 2014) and maize (Dorner *et al*., 1999). Influences of atoxigenic biocontrol products extend beyond the season of treatment as a result of survival of the atoxigenic active ingredients in soils (Cotty *et al*., 2007; Bandyopadhyay *et al*., 2016). Recently, biocontrol technology has been adapted to reduce aflatoxin in maize and groundnut in Kenya and several other African nations through a partnership between the International Institute of Tropical Agriculture (IITA), the USDA‐Agricultural Research Service (USDA‐ARS) and several National Agricultural Research Services including the Kenya Agricultural and Livestock Research Organization (KALRO) (Bandyopadhyay and Cotty, 2013; Bandyopadhyay *et al*., 2016).

*Aspergillus flavus* is commonly divided into two strains or morphotypes, the L‐strain morphotype and the S‐strain morphotype, which differ in not only morphology but also physiology, genetics and consistency of aflatoxin production (Cotty, 1989). Almost all members of the S‐strain morphotype produce high levels of aflatoxins (Cotty, 1989); however, atoxigenic strains of the L‐strain morphotype are frequent in crops and soils (Cotty, 1990, 1994a, 1994b, 1997,1990, 1994a, 1994b, 1997). Many of these atoxigenic strains are effective as biocontrol agents for aflatoxin management (Mehl *et al*., 2012; Bandyopadhyay and Cotty, 2013; Atehnkeng *et al*., 2014; Ehrlich, 2014; Bandyopadhyay *et al*., 2016; Agbetiameh *et al*., 2019; Moral *et al*., 2020).

Atoxigenic *A. flavus*‐based aflatoxin biocontrol products directed at various regions in Africa are named Aflasafe™ with a country‐specific suffix. Each Aflasafe product contains atoxigenic strain active ingredients endemic to the target nation. Aflasafe KE01™ (hitherto called KE01) is registered with the Pest Control Products Board (PCPB) of Kenya as a Kenya‐specific aflatoxin biocontrol product. This product contains four distinct atoxigenic *A. flavus* L‐morphotype active ingredients (C6E, E63I, R7H and R7K; Bandyopadhyay and Cotty, 2013; Bandyopadhyay *et al*., 2016; Moral *et al*., 2020). These active ingredients were isolated from maize produced in Kenya from 2004 to 2006 with the intent of selecting effective fungi well adapted to maize agroecosystems in Kenya (Probst *et al*., 2011). However, natural distributions of these selected genotypes in Kenyan agroecosystems beyond the grain from which they were isolated are unknown. Data on distribution of KE01 active ingredients prior to initiation of commercial treatments are necessary to quantify extents of long‐term, cumulative benefits in treated regions of Kenya.

Natural populations of *A. flavus* are composed of hundreds of vegetative compatibility groups (VCGs) (Bayman and Cotty, 1991; Cotty *et al*., 1994). Vegetative compatibility groups are defined by a heterokaryon incompatibility system that allows anastomosis and gene flow among closely related genotypes but restricts anastomosis among dissimilar individuals (Grubisha and Cotty, 2010, 2015). *Aspergillus flavus* VCGs evolve as clonal lineages (Papa, 1986; Leslie, 1993; Ehrlich *et al*., 2007; Grubisha and Cotty, 2010). Vegetative compatibility analyses (VCA) are used to track and characterize *A. flavus* active ingredients (Bayman and Cotty, 1993; Cotty, 1994a, 1994b,1994a, 1994b; Atehnkeng *et al*., 2014; Agbetiameh *et al*., 2020; Senghor *et al*., 2020). However, VCA are laborious culture‐based techniques, and each VCG must be assayed independently with the workload multiplying by the number examined. Vegetative compatibility analyses also require maintenance of living, functional tester mutants for each VCG (Bayman and Cotty, 1993; Horn *et al*., 1996; Probst *et al*., 2011), and these mutants must be shared among laboratories wishing to identify or monitor active ingredients. In this regard, DNA technologies can be advantageous (Shenge *et al*., 2019).

Simple sequence repeats (SSR) are effective at differentiating genotypes of *A. flavus* (Grubisha and Cotty, 2015; Picot *et al*., 2018; Senghor *et al*., 2020). Fungal VCGs frequently contain multiple closely related SSR haplotypes (Berbegal *et al*., 2010; Chang *et al*., 2014). SSR markers are valuable tools for genotyping *A. flavus* from agricultural (Tran‐Dinh *et al*., 2009; Grubisha and Cotty, 2010, 2015; Sweany *et al*., 2011; Ortega‐Beltran *et al*., 2020) and clinical settings (Hadrich *et al*., 2010; Dehghan *et al*., 2014).

In the present study, 17‐SSR loci distributed across all eight chromosomes of *A. flavus* were used to determine distributions of the four active ingredients of KE01 in soils under maize cultivation in seven counties across Kenya. An objective of this study was to determine relationships of the active ingredient SSR haplotypes to VCA used to monitor the active ingredients in Kenya. In addition, this work sought to characterize natural frequencies and distributions across fungal communities in Kenyan agroecosystems with high incidences of aflatoxin contamination prior to initiation of aflatoxin management programmes based on the biocontrol product. During the process, genetic lineages containing each of the active ingredients were characterized.

## Results

### Diversity of potential aflatoxin‐producing *Aspergillus* section *Flavi*

**Fig. 1:**
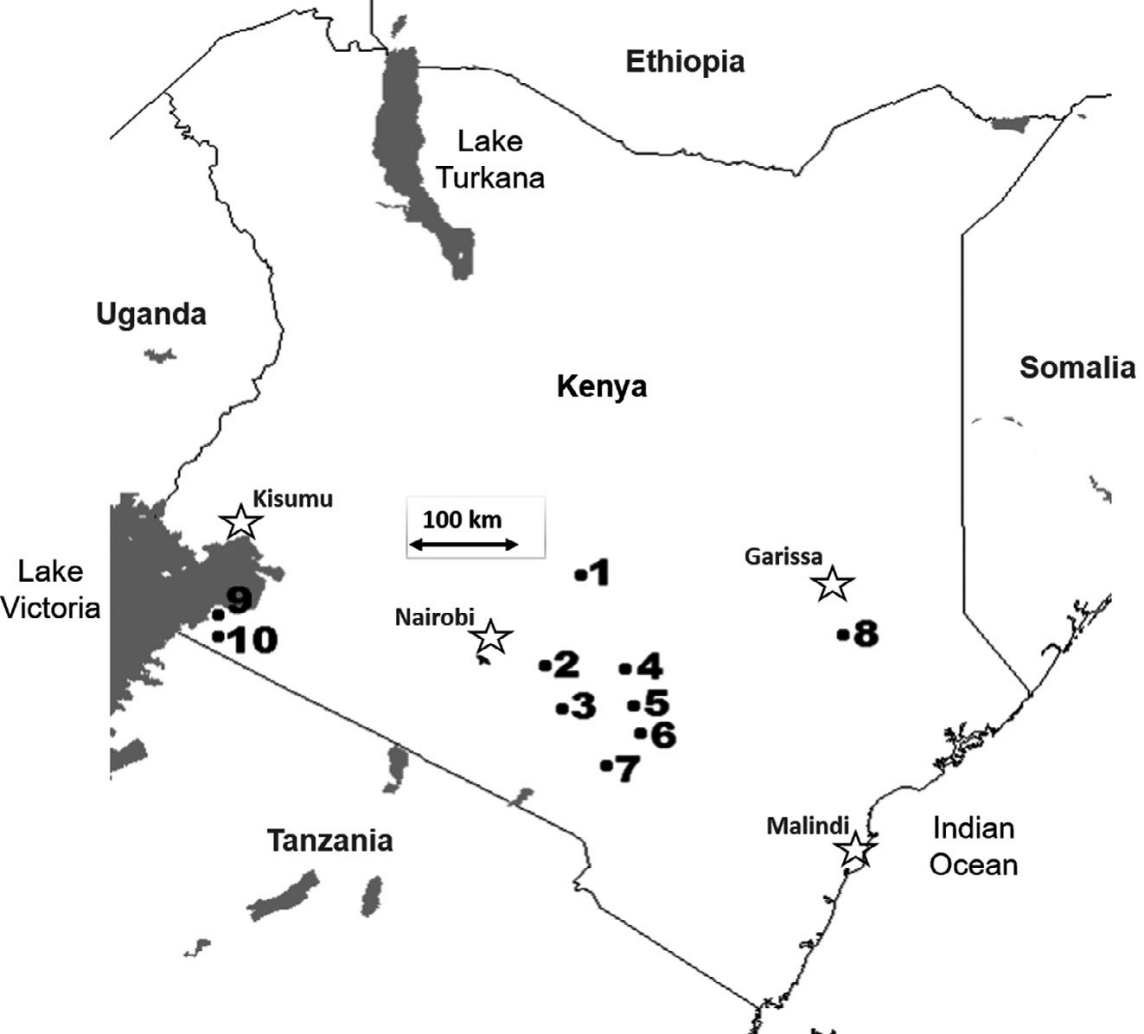
Map of Kenya. Numbers indicate locations of 10 ten agricultural areas from seven counties in Kenya (corresponds to Table 1) in which soils were sampled. These are representative of the primary target areas for which the aflatoxin biocontrol product was developed.

A total of 7189 *Aspergillus* section *Flavi* fungal isolates were recovered from 629 maize field soils during the long rains and the short rains seasons of 2012. Sampled fields spanned 10 agricultural areas in seven counties (Fig. 1). The fungi included 2812 isolates from *A. flavus* L‐strain morphotype, 3,400 isolates of LAF (LAF = Lethal Aflatoxicosis Fungus; Kachapulula *et al*., 2017; Singh *et al*., 2020) from a high aflatoxin‐producing unnamed species with S morphology associated with lethal aflatoxin contamination of maize produced in Kenya (Probst *et al*., 2007; Probst *et al*., 2012) and 986 *A. parasiticus* (Table 1). Each of these species was detected in all ten of the sampled agricultural areas across Kenya. However, LAF and the L morphotype of *A. flavus* were the most common. Similar frequencies of LAF occurred in all areas except Area‐8 (Tana River County), where LAF was rarely detected (Table 1).

**Table 1:** *Aspergillus* section *Flavi* fungal isolates recovered from maize field soils from ten agricultural areas in seven counties in Kenya during the 2012 long rains and short rains seasons.

Sampling details			Long rains season (May 2012)						Short rains season (November 2012)			
Agricultural area	District/location	County	Soils^a^ (#)	Isolates^a^ (#)	*A. flavus* ^b^ (#)	LAF^c^ (#)	*A. parasiticus* ^d^ (#)	Soils^a^ (#)	Isolates^a^ (#)	*A. flavus* ^b^ (#)	LAF^c^ (#)	*A. parasiticus* ^d^ (#)
Area‐1	Embu East; Mbeere North	Embu	121	1445	368	892	185	60	643	169	445	29
Area‐2	Kangundo; Kathiani; Matungulu	Machakos	63	749	300	291	158	32	340	112	131	97
Area‐3	Machakos; Makueni; Mbooni East	Machakos; Makueni	93	1118	312	690	116	70	725	187	385	153
Area‐4	Kitui Central; Nzambani	Kitui	40	470	185	202	83	31	335	153	132	50
Area‐5	Mutomo	Kitui	–	–	–	–	–	10	110	86	23	1
Area‐6	Ikutha	Kitui	14	174	149	22	3	10	102	77	23	2
Area‐7	Makindu	Makueni	19	241	160	63	18	10	100	36	57	7
Area‐8	Tana River	Tana River	40	471	465	1	5	–	–	–	–	–
Area‐9	Homabay	Homabay	–	–	–	–	–	8	89	31	38	20
Area‐10	Rongo	Migori	–	–	–	–	–	8	86	22	5	59
Total (10 Areas, 16 locations; 7 Counties)			390	4668	1939	2161	568	239	2530	873	1239	418

^a^ Number of fields sampled. A single composite soil sample composed of 30 to 40 subsamples along a 40 to 50 m transect was taken from each field.

^b^ Total number of total isolates of *Aspergillus* section *Flavi* (*A. flavus*, LAF and *A. parasiticus*) isolates recovered.

^c^ Number of isolates identified as *Aspergillus flavus* L morphotype.

^d^ Number of isolates belonging to the Kenyan S morphotype referred to as the Lethal Aflatoxicosis Fungus (LAF; Kachapulula *et al*., 2017; Singh *et al*., 2020) which has been associated with contamination events that led to deaths in Kenya (Probst *et al*., 2012, 2007).

^e^ Number of isolates identified as *Aspergillus parasiticus*.

### SSR amplification, genotyping and clone correction

A total of 2744 isolates were clearly amplified at 17‐SSR loci and included in this analysis (Table 2). SSR amplification across these isolates was free of PCR artefacts and only generated single peaks in the expected size range. There were no missing data and no null alleles. The number of alleles per locus was high and ranged from 9 to 72. High allelic diversity resulted in high genotypic diversity, with 2140 unique SSR haplotypes (Table 2).

**Table 2:** Genetic diversity of *A. flavus* L‐morphotype isolates in 629 maize field soils across ten agricultural areas from seven counties in Kenya during 2012.

Area^a^	District/location	County	Total isolates^b^	SC isolates^c^	Haplotypes^d^
1	Embu East; Mbeere North	Embu	519	479	453
2	Kangundo; Kathiani; Matungulu	Machakos	389	341	326
3	Machakos; Makueni; Mbooni East	Machakos; Makueni	490	420	383
4	Kitui Central; Nzambani	Kitui	322	295	281
5	Mutomo	Kitui	86	78	77
6	Ikutha	Kitui	224	192	188
7	Makindu	Makueni	187	154	149
8	Tana River	Tana	474	402	356
9	Homabay	Homabay	31	27	27
10	Rongo	Migori	22	20	20
Total			2744	2408	2140

^a^ Area locations are illustrated in Fig. 1.

^b^ Total number of isolates analysed.

^c^ Number of isolates after sample correction (removal of isolates with repeated haplotypes in the same soil sample).

^d^ Total number of SSR haplotypes.

Isolates were culled to only allow a single isolate with any given SSR haplotype from each soil. After this clone correction, 2408 isolates remained for analysing frequencies, distribution, adaptation and lineage relationships of the four KE01 active ingredients within naturally occurring fungal populations in Kenyan soil.

### Relationships between SSR haplotype and VCG of KE01 isolates

The four KE01 active ingredients (C6E, E63I, R7H and R7K) were separated by both VCG analysis and SSR typing (Table 3). Haplotypes of 24 soil isolates matched the R7K haplotype, and *nit* ^‐^ mutants of each of these isolates complemented an auxotrophic tester mutant of R7K but not any of the six tester mutants developed for the other three KE01 active ingredients. Complementation of an R7K tester mutant indicates membership in the R7K VCG. In a similar manner, all isolates matching the haplotypes of C6E (*n* = 15), E63I (*n* = 3) and R7H (*n* = 10) were shown to belong to the same VCG as the respective active ingredient. In addition, isolates differing from C6E (*n* = 5), E63I (*n* = 13) and R7H (*n* = 3) at only a single SSR locus belonged to the same VCG as the nearly‐matching active ingredient (Table 3). In no case did any of the active ingredients react with a tester mutant developed from a different active ingredient.

**Table 3:** Relationship of vegetative compatibility group (VCG) to identical and closely related haplotypes of Aflasafe KE01 active ingredients.

Aflasafe KE01 active ingredient^b^	Haplotype ID	No. of isolates	SSR loci and alleles^a^																	Mutational changes (distance) from KE01 Haplotype	VCG ID^b^
			AF28	AF 13	AF 43	AF 22	AF 31	AF 42	AF 8	AF 53	AF 34	AF 16	AF 54	AF 17	AF 11	AF 66	AF 64	AF 63	AF 55		
R7K	H‐1462	24	135	155	385	192	352	159	171	134	301	169	161	359	123	275	191	127	180	0	KN012
C6E	H‐0199	15	113	141	379	192	315	159	171	134	320	169	161	359	159	279	169	129	184	0	KN00A
	H‐0197	3	113	141	379	192	315	159	171	134	320	169	161	359	159	277	169	129	184	1 (One step)	KN00A
	H‐0198	1	113	141	379	192	315	159	171	134	320	169	161	359	159	279	169	129	172	1 (Six steps)	KN00A
	H‐0200	1	113	141	379	192	315	159	171	134	320	169	161	359	162	279	169	129	184	I (One step)	KN00A
E63I	H‐1019	3	131	135	379	196	361	181	215	134	301	169	172	359	123	257	169	133	178	0	KN001
	H‐1017	13	131	135	379	196	361	181	215	134	301	169	172	359	123	255	169	133	178	1 (One step)	KN001
R7H	H‐0212	10	113	141	379	196	361	181	209	134	320	178	172	359	159	253	227	129	178	0	KN011
	H‐0214	3	113	141	379	196	361	181	209	134	320	178	172	359	159	255	227	129	178	1 (One step)	KN011

^a^ The 17 SSR loci are named (AF‐28 to AF‐55). Allele sizes indicate amplicon size in base pairs as called on an ABI 3730 DNA Analyzer with the LIZ500 standard (Applied Biosystems).

^b^ Aflasafe KE01 active ingredients registered for aflatoxin mitigation in Kenya. Active ingredient names and VCG names are from Probst *et al*. (2011).

### Distributions of KE01 haplotypes

In total, 118 haplotypes occurred in 2 to 23 soil samples (data not shown). The haplotypes of each of the four KE01 active ingredients were among the first ten most frequently occurring haplotypes (Table 4). These haplotypes occurred in multiple areas and both seasons. The haplotype of active ingredient R7K (H‐1462) was detected in 3.8% (23 total) of soils and was the most widespread haplotype (occurring in eight of ten study areas) in both long rains and short rains seasons (Table 4). Haplotypes belonging to two other active ingredients (C6E and R7H) were found in 2.4% and 1.8% of the soils, respectively, and were both found in five areas and both seasons (Table 4). H‐1019, the haplotype of the fourth KE01 (E63I), was detected in only 3 soils (0.6%) from two areas. However, H‐1019 differs from H‐1017 at only a single locus and at that locus by only a single SSR repeat. H‐1017 belongs to the same VCG as H‐1019, and H‐1017 was the fourth most commonly detected haplotype and occurred in 13 soils from multiple areas and both seasons (Tables 3 and 4).

**Table 4:** Distributions of the ten most frequent *Aspergillus flavus* L‐morphotype haplotypes identified in soils from Kenya. Haplotypes of all four Aflasafe KE01 active ingredients are included.

Haplotype ID^a^	N^b^	Area‐1^c^		Area‐2		Area‐3		Area‐4		Area‐5	Area‐6		Area‐7		Area‐8	Area‐9	Area‐10
		S1^d^	S2^e^	S1	S2	S1	S2	S1	S2	S2	S1	S2	S1	S2	S1	S2	S2
H‐1462 (R7K)^a^	**23**	**3**	**3**	**3**	**1**	**1**	**4**	0	**3**	**2**	0	**1**	**1**	0	0	1	0
H‐1354	**17**	0	**2**	**2**	**1**	**1**	**6**	0	**2**	0	0	**1**	0	0	1	0	**1**
H‐0199 (C6E)^a^	**14**	0	**2**	0	**3**	**1**	**2**	**1**	**4**	0	0	0	0	0	0	0	**1**
H‐1017	**13**	0	**2**	1	**1**	0	**5**	0	**3**	0	0	**1**	0	0	0	0	0
H‐0212 (R7H)^a^	**10**	0	**1**	0	**1**	0	**4**	**1**	**2**	0	0	**1**	0	0	0	0	0
H‐0591	**8**	0	0	0	0	**1**	0	**1**	0	**1**	**1**	0	**3**	0	**1**	0	0
H‐0318	**6**	**1**	0	0	0	**1**	0	0	**1**	0	0	0	**2**	0	**1**	0	0
H‐0557	**5**	0	**3**	0	0	0	**1**	0	0	0	0	0	0	0	**1**	0	0
H‐1158	**4**	**1**	0	**1**	0	**1**	0	0	0	0	0	**1**	0	0	0	0	0
H‐1019 (E63I)^a^	**3**	**1**	0	0	0	0	**2**	0	0	0	0	0	0	0	0	0	0

^a^ Haplotype of one of the four active ingredients of Aflasafe KE01, a registered biopesticide for aflatoxin management in Kenya. H = haplotype; unique haplotypes were numbered H0001 to H2710. Haplotypes were identified based on 17‐multilocus SSR loci. Only the ten most common haplotypes across both seasons and all areas are included. Numbers in columns indicate the number of isolates with the indicated haplotype in each area (Area 1 to 10) and season (S1 & S2).

^b^ *N* = total number of isolates overlapped with same haplotypes.

^c^ Locations Area 1 through Area 10 are indicated in Fig. 1.

^d^ S1, Long Rain Season (May 2012).

^e^ S2, Short Rain Season (November 2012).

### Distribution of closely related lineages of Aflasafe™ KE01

**Fig. 2:**
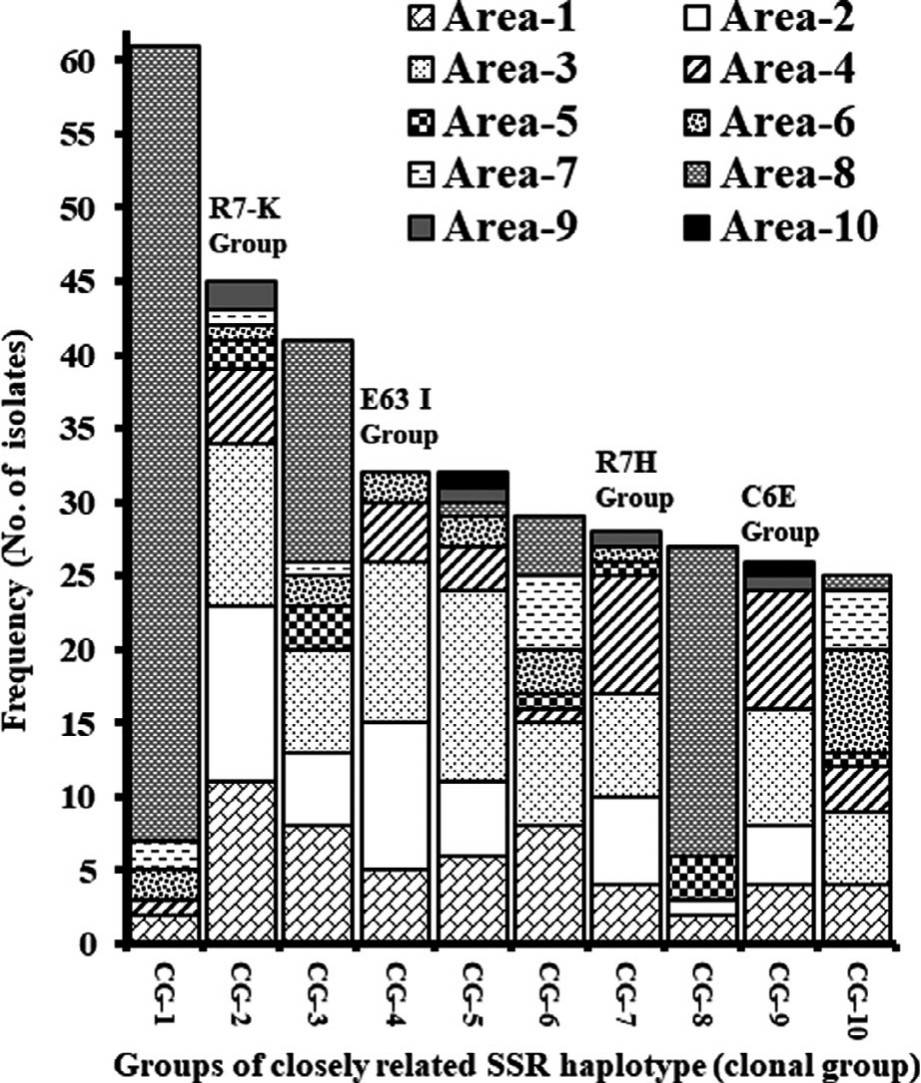
The 10 most frequently occurring clonal groups (CGs) of closely related haplotypes (sharing identical genotypes for at least 14 of the 17 SSR loci) of *A. flavus* including the groups of the four Aflasafe KE01 active ingredients (indicated with active ingredient name on top of the bars). Locations of the 10 agricultural areas (named Area‐1 through Area‐10) are indicated in Fig. 1. *A. flavus* populations were sampled in maize soils in both the long rains and short rains seasons during 2012. The results above combine the data of the two seasons.

The eBurst analysis identified 246 groups (clonal lineages) of closely related haplotypes based on a user‐defined criteria (differing at no more than 3 of 17 loci and > 80% genetic similarity). The four KE01 isolates were each included in the top ten largest clonal groups containing 26 to 45 haplotypes distributed across multiple areas (7 to 9 areas; Fig. 2). However, no member of any of the clonal groups of the four KE01 isolates was found in Area‐8 (Fig. 2). The clonal groups of R7K, C6E, R7H and E63I were found in 7.2%, 4.1%, 4.5% and 5.1% of the soil samples respectively (Fig. 2). In total, clonal lineages of the active ingredients occurred within 21% of examined soils.

## Discussion

The current results agree with previous observations that the *A. flavus* L morphotype is widely distributed in agricultural soils across Africa and including Kenya (Donner *et al*., 2009; Probst *et al*., 2014; Table 1). Populations of S‐morphology fungi in Africa and North America are distinct (Cotty and Cardwell, 1999). Although the *A. flavus* S morphotype is common in the USA (Singh *et al*., 2020), it either very rare or does not occur in Kenya (Probst *et al*., 2012, 2007). However, there are common S‐morphology fungi in Kenya, sometimes referred to as LAF (LAF = Lethal Aflatoxicosis Fungus; Kachapulula *et al*., 2017; Singh *et al*., 2020) that are phylogenetically distinct from *A. flavus* but still consistently produce high concentrations of aflatoxins in maize and were associated with lethal aflatoxicoses occurring in Kenya from 2004 to 2006 (Probst *et al*., 2014, 2012). The LAF is common in regions with histories of lethal aflatoxicosis, including Machakos, Makueni and Kitui counties (Probst *et al*., 2012, 2007, 2010). In the current study, LAF was only infrequent in Area‐8 (Tana River County; Table 1) which is primarily the Tana River Irrigation Scheme near the town of Bura. Another high aflatoxin‐producing species, *A. parasiticus,* composed more than 10% of the aflatoxin producers in four areas but was also not detected in Area‐8. *A. parasiticus* and LAF may not be well adapted to the agroecology of Area‐8, the only area where maize is produced primarily under furrow irrigation. Factors dictating geographic distributions of LAF and *A. parasiticus* need further investigation. Where these fungi occur, applications of KE01 are expected to alter their frequencies downwards. The current data provide baselines with which to assess extents to which such alterations occur across Kenya.

The observed high allelic diversity (Table 2) resulted in a d over 2000 haplotypes across the study populations. This indicates an exceptionally large population in Kenya soils because SSR allelic and haplotype diversity is a function of population size, stability and evolution (Islam *et al*., 2018). High diversity of the *A. flavus* population in Kenya soil can also be measured with VCG diversity (Bayman and Cotty, 1991, 1993; Atehnkeng *et al*., 2016; Ortega‐Beltran and Cotty, 2018). *A. flavus* VCGs in natural populations can evolve independently for long periods (Ehrlich *et al*., 2007; Grubisha and Cotty, 2010), and each VCG can be considered an individual genetic lineage delineated by multiple heterokaryon incompatibility loci (Leslie, 1993). Multiple VCGs may occur within a single seed or pinch of soil (Bayman and Cotty, 1991; Mehl and Cotty, 2013). Diversity of *A. flavus* VCGs in soil populations is high in established agricultural settings in warm climates (Bayman and Cotty, 1991; Horn, 2003), including Kenya (Probst *et al*., 2011). It is expected that there are fewer VCGs than SSR haplotypes because each VCG typically contains multiple, closely related SSR haplotypes (Grubisha and Cotty, 2010; Ortega‐Beltran and Cotty, 2018).

*Aspergillus flavus* is primarily recognized as an asexual fungal species. Under clonal reproduction, identical haplotypes displayed by isolates from two or more soils (repeated haplotypes) are expected. Although a large number of unique haplotypes were detected because of the exceptionally large and ancient population of *A. flavus* in Kenya soils (Islam *et al*., 2018), only 118 haplotypes occurred in two or more soils (data not shown). This relatively low number of repeated haplotypes may be a result of how difficult it is to thoroughly sample such a diverse population. Each of the four active ingredients of the biocontrol product has a distinct SSR haplotype and a distinct VCG. Results from the SSR assay were in complete agreement with VCG analyses used to track other atoxigenic *A. flavus* and, as such, this assay is also a reliable tool (Cotty, 1994a, 1994b,1994a, 1994b; Atehnkeng *et al*., 2014; Ortega‐Beltran and Cotty, 2018; Agbetiameh *et al*., 2019). Vegetative compatibility groups are clonal lineages (Papa, 1986; Leslie, 1993), and linkage equilibrium of SSR loci occurs among isolates within the same VCG, possibly indicating an active parasexual cycle, but not among isolates in different VCGs (Grubisha and Cotty, 2010; Grubisha and Cotty, 2015; Ortega‐Beltran *et al*., 2020). Over long periods, mutation and clonal evolution may cause diversity at SSR loci within VCGs (Islam *et al*., 2018). This is evident in the current data set with three of the active ingredients. The VCGs of those active ingredients include one to three haplotypes different at one SSR locus (Table 3). Indeed, the fourth most common haplotype belongs to the VCG of active ingredient R7K, again suggesting better adaptation to Kenya’s agroecosystems than most VCGs not included in KE01.

Soil collections for this study were undertaken 6–7 years after harvest of the maize from which the active ingredients of the biocontrol product were collected (Probst *et al*., 2011) indicating persistence of these clonal lineages over long periods. The high frequencies, wide distributions (Table 4) and long persistence of the KE01 haplotypes and their close relatives (clonal lineages) across the study areas suggest that these genotypes are well adapted to Kenyan agroecosystems. Although haplotypes of the KE01 active ingredients are relatively common, and natural presence of these atoxigenic genotypes likely positively influence crop contamination (Cotty *et al*., 2008), the background levels of these genotypes is far below what would be required to prevent unhealthy levels of aflatoxins. Therefore, biocontrol treatments with KE01, which are expected to result in over 80% frequencies of the active ingredients on the crop (Bandyopadhyay *et al*., 2016), will likely provide benefit. The current work provides reference points from which to judge both single‐season and long‐term efficacies across portions of Kenya most in need of aflatoxin mitigation.

Certain VCGs and SSR haplotypes of *A. flavus* are naturally widely distributed. For instance, several VCGs of *A. flavus* isolated from agricultural soils occur across a large section of the United States (Horn and Dorner, 1998, 1999; Ehrlich *et al*., 2007; Grubisha and Cotty, 2010, 2015; Ogunbayo *et al*., 2013) and others occur across Africa (Ogunbayo *et al*., 2013). Dispersal of *A. flavus* is consistent with the production of large quantities of airborne conidia (Bock *et al*., 2004) and association with both insects (Stephenson and Russell, 1974) and human transported crop materials (Garber and Cotty, 2014; Shenge *et al*., 2019). Detection of the KE01 active ingredients in all areas except Area‐8 (Tana River County) of the Tana River Irrigation Scheme near Bura is most likely due to either physical barriers to dispersal (i.e. stretches of arid land) or requirements for adaptive success differing among areas. Different requirements for niche residence in Area‐8 may cause high frequencies of locally restricted haplotypes (Islam *et al*., 2018). In some cases, production areas with distinct agroecologies or aflatoxin aetiology may warrant the development of targeted biocontrol agents.

In conclusion, high genotypic diversity in the *A. flavus* population resident in Kenya is attributable to the size (very large) and age (ancient) of that population (Islam *et al*., 2018). The wide distribution and persistence of SSR haplotypes closely related to the four active ingredients of KE01, especially 6–7 years after initial isolation, indicate that these fungal genotypes and genetic lineages have been growing in and adapting to Kenyan agroecosystems for long periods. Thus, the KE01 active ingredients can be expected to perform and compete well against resident aflatoxin producers. The results of the current study suggest that these active ingredients are excellent selections for a Kenya‐specific aflatoxin biocontrol product for which long‐term efficacy, persistence and cumulative benefits are desired.

## Experimental procedures

### Sample collection

Soils from agricultural fields cropped to maize were sampled in seven counties (Embu, Machakos, Makueni, Kitui, Tana, Homabay and Migori) located across the regions of Kenya most vulnerable to aflatoxin contamination, as previously described (Islam *et al*., 2018). Sampling was performed during both the long rains (late April to Early June) and the short rains (November to December) seasons of 2012 (Table 1). The two seasons are separated by approximately six months. Five counties (Tana River, Makueni, Kitui, Machakos and Embu) were sampled just prior to the long rains season (May 2012), and six counties (Embu, Machakos, Makueni, Kitui, Homabay and Migori) were sampled just prior to the short season rains (November 2012) (Table 1). Fields were geo‐referenced prior to sampling. Samples were collected from 629 fields across ten agricultural areas (Fig. 1), where 390 fields were sampled during the long rains season and 239 fields during the short season rains (Fig. 1 and Table 1) following a previously described method (Cotty, 1997). Briefly, in each field, a 30 to 40 m transect was sampled at 3‐ to 4‐m intervals. At each point, four small scoops of soil (1 to 3 g each) were collected to a depth of 2 cm and combined with samples from other points. These pooled samples were homogenized and stored dry at room temperature. All samples were shipped to the USDA‐ARS laboratory at the School of Plant Sciences of the University of Arizona, Tucson, under a permit from USDA‐APHIS (permit to move live plant pests, noxious weeds, and soil). The active ingredients of KE01 (C6E, E63I, R7H and R7K) belong to the L morphotype and were originally isolated in the USDA‐ARS laboratory at the University of Arizona from maize produced in Kenya during the lethal aflatoxicosis outbreaks of 2004 through 2006 (Probst *et al*., 2011).

### Isolation of *Aspergillus* section *Flavi* fungi

Soil samples were subjected to the dilution plate technique on the semi‐selective medium modified Rose Bengal agar (M‐RB) (Cotty, 1994a, 1994b,1994a, 1994b). Briefly, 5 to 10 g of soil was suspended in 250 ml sterile distilled water with 0.1% TWEEN‐80 solution by stirring for 20 min at 200 rpm. Aliquots of the resulting suspension were spread on three M‐RB plates (200 µl per plate). When necessary, adjustments to aliquot volume and soil quantity were made to obtain no more than 12 *Aspergillus* section *Flavi* colonies per plate. After incubation at 31°C for three days, *Aspergillus* section *Flavi* colonies were subcultured onto 5/2 agar [(5% V‐8 juice (Campbell Soup Company, Camden, NJ), 20 g l^−1^ bacto‐agar, pH 6.0] at 31°C (Cotty, 1989). A total of 10 to 15 *Aspergillus* section *Flavi* isolates were cultured from each soil sample, and specific fungal species or morphotypes were identified on the basis of colony characteristics and spore morphology. All isolates belonging to the *A. flavus* L morphotype (Table 1) were included for further analyses. Cultures were stored as 3‐mm plugs of 5‐day‐old cultures on 5/2 agar in sterile distilled water at 4°C for further use. For long‐term storage, select cultures were stored on silica gel. The active ingredients (C6E, E63I, R7H and R7K) of KE01 were cultured from silica storage maintained at the Tucson location. One piece of silica was subcultured on 5/2 agar as previously (Cotty, 1989). Sporulating cultures (5–7 days old) were stored as 3‐mm plugs on 5/2 agar in sterile distilled water at 4°C until further use.

### DNA extraction and SSR amplification

DNA was extracted from active ingredients (C6E, E63I, R7H and R7K) of KE01, and all *A. flavus* L‐morphotype isolates recovered from soil samples (Table 1). A previously described protocol was followed for DNA extraction (Callicott and Cotty, 2015). Briefly, isolates were plated onto 5/2 agar (Cotty, 1989) and incubated at 31°C for 8–10 days. Conidia were then suspended in 500 µl lysis buffer and incubated with agitation in a Thermomixer 5436. DNA was ethanol precipitated, dissolved in sterile water, quantified with a spectrophotometer (Nanodrop, Wilmington, DE) and stored at −20°C until use.

Variability was determined at 17 SSR loci distributed across all eight *A. flavus* chromosomes, as previously described (Islam *et al*., 2018). Briefly, five multiplex PCR panels optimized for high‐throughput SSR amplification and genotyping. Multiplex PCRs were carried out using 0.08 µmol l^−1^ of each primer, 1X AccuStart II PCR SuperMix (Quanta Biosciences, Gaithersburg, MD, USA) and 5 ng of genomic DNA in a final reaction volume of 10 µl. PCR conditions were as follows: 94°C for 1 min, 26 cycles of 94°C for 30 s, 57°C for 90 s, 72°C for 30 s and 30 min at 60°C. PCR amplicons were sized at the University of Arizona Core Genomics Laboratory with the LIZ500 standard and an ABI 3730 analyzer (Applied Biosystems, Waltham, Massachusetts, USA). Allele sizes were called with genemarker version 2.6 (SoftGenetics LLC, State College, Pennsylvania, USA).

### SSR genotyping and clone correction

Allelic data from 17‐SSR markers were combined for each of the study isolates, and haplotypes (genotypes) were determined using the program HAPLOTYPE‐ANALYSIS V 1.04 (Eliades and Eliades, 2009). In order to construct a clone‐corrected data set for each sample, each haplotype was only included in the dataset once for each soil sample (Table 2). Clone‐corrected data sets were used as the basis for further analysis.

### Analysis of vegetative compatibility group (VCG)

In order to test whether isolates with an SSR haplotype matching one of the active ingredients of KE01 belonged to that active ingredient’s VCG, isolates were typed using VCA based on complementation of nitrate non‐utilizing auxotrophs (Bayman and Cotty, 1991). All isolates with haplotypes either matching an active ingredient of KE01 or closely related to an active ingredient’s haplotype (differing at only one SSR locus) were typed with VCA. Briefly, isolate auxotrophs (*nit* ^–^ mutants) were generated for each isolate on chlorate containing media (Cotty, 1994a, 1994b,1994a, 1994b). Complementary tester auxotrophic mutants (one *cnx^–^* and one *niaD^‐^*) from each of the four active ingredients of KE01 were previously developed (Probst *et al*., 2011). A nitrate auxotroph (*nit* ^‐^) from each isolate was paired with both tester auxotrophs. Isolate auxotrophs that complemented one or both tester auxotrophs were classified as belonging to the VCG defined by that tester pair.

### Analysis of genotypic diversity and distributions

Haplotypic diversity, frequencies and distributions were estimated for both cropping seasons (long and short rains) for the year 2012, both individually and together. Area‐wide haplotypic diversity, including the number of haplotypes and frequencies of repeated haplotypes displayed by two or more isolates, was measured using the program haplotype‐analysis V 1.04. Frequencies of the most common haplotypes across the study areas were statistically tested with analysis of variance (ANOVA) based on the general linear model procedure in sas (version 9.2; SAS Institute, Cary, NC). Mean separations were performed on data from tests with statistically significant differences (*P* = 0.05) using Tukey’s honestly significant difference test (Pagano and Gauvreau, 2000).

### Analysis of closely related lineages of the biocontrol product

Closely related lineages (closely related haplotypes are presumed to have evolved through clonal reproduction and mutation) of the active ingredients of KE01 were identified based on principles used for determining clonal lineages of the asexually reproducing bacteria with the program eburst v3 (http://eburst.mlst.net/ ) (Feil *et al*., 2004). A user‐defined group definition was set to include those groups that shared identical genotypes for at least 14 of the 17 SSR loci.

## Conflict of interest

All authors declare no conflict of interest.
